# Evaluation of human body malodor using functional near-infrared spectroscopy with regions of interest based on functional pathways in the olfactory system

**DOI:** 10.3389/fnins.2026.1808067

**Published:** 2026-05-05

**Authors:** Yoshihiro Inoue, Kaori Osaki, Satoshi Yomota, Ayumi Kyuka, Yuka Kyuma, Ayaka Yoshimoto, Takeshi Hara, Hidefumi Ikeda

**Affiliations:** 1 Analytical and Measuring Instrument Division, Shimadzu Corporation, Kyoto, Japan; 2 Advanced Technology Institute, Mandom Corporation, Osaka, Japan; 3 Laboratory of advanced Cosmetic Science, Graduate School of Pharmaceutical Sciences, The University of Osaka, Osaka, Japan

**Keywords:** functional near-infrared spectroscopy (fNIRS), functional pathway, human body odor, malodor, olfactory system, oxygenated hemoglobin, region of interest, stress marker

## Abstract

**Introduction:**

Malodors in the human body can diminish comfort in living and working environments, potentially increasing stress and dissatisfaction levels. Therefore, there is a growing need to objectively characterize the properties of human body malodor. The brain extracts various types of information from odor stimuli. Although the central processing pathways of the human olfactory system are not fully understood, some suggest that it comprises distinct parallel functional pathways. This study aimed to clarify whether human body malodor can be objectively evaluated using functional near-infrared spectroscopy (fNIRS) by defining regions of interest in the cerebral cortices based on functional pathways in the olfactory system.

**Materials and methods:**

Eighteen healthy, right-handed Japanese adults in their 40s (nine women and nine men) participated in this study. During the three odor stimuli (middle-aged female scalp model odor (MFS model odor), isovaleric acid, and lavender), fNIRS measurements were performed on the cerebral cortical areas connected to the parallel functional pathways in the olfactory system. Odor intensity was adjusted to allow continuous inhalation without inducing marked changes in mood or stress, as confirmed by answering a questionnaire on temporary mood scales and measuring stress markers (heart rate, nose-tip temperature, fingertip blood flow, salivary cortisol, and salivary alpha amylase).

**Results:**

The MFS model odor elicited greater increases in oxygenated hemoglobin concentrations than lavender in the inferior frontal gyrus and left superior temporal gyrus, both of which are cortical regions that receive projections from the temporal piriform cortex. These differences in neural responses ceased to be significant after adjusting for perceived intensity, pleasure/displeasure valence, or subjective preference ratings. No significant odor-dependent differences were detected in physiological or endocrine stress markers.

**Conclusion:**

This study demonstrates the feasibility of objectively evaluating human body malodor using fNIRS in relation to cortical processing via olfactory pathways. The findings suggest that neural responses to human body odor, particularly in language-related cortical regions, do not simply reflect odor category, but are closely associated with subjective perceptual evaluations. This approach enables objective assessment of human body odor and holds potential for applications in sensory science and product development.

## Introduction

Odors are related to emotions and memories and impact human physiology, psychology, and behavior in daily life (Krusemark et al., 2013; Walla et al., 2003; Walla, 2008). Understanding the effects of odors on humans is of academic interest and is important from the perspective of industrial and clinical applications. Odor is an important element in cosmetics. This applies to both pleasant and unpleasant odors. Human body malodors can reduce comfort in living spaces and workplaces and increase stress and dissatisfaction. Because numerous people suffer from body malodor, it is necessary to understand the various characteristics of human body malodors (Mitro et al., 2012; Haze et al., 2015; Son et al., 2025).

Subjective measures, such as questionnaires, are frequently used to evaluate the effects of odors on humans. However, because subjective evaluations are susceptible to cognitive biases and other limitations (Dalton et al., 1997; Yamanaka et al., 2009), research has increasingly focused on obtaining additional insights using objective assessment methods. Objective data include physiological indicators such as heart rate and measures of brain function obtained through neuroimaging techniques, including functional magnetic resonance imaging (fMRI) and functional near-infrared spectroscopy (fNIRS). fMRI, which leverages blood oxygenation level–dependent contrast, enables high spatial resolution mapping of neural activity (Ogawa et al., 1990). However, its complex operation and high associated costs limit its widespread application.

To overcome these limitations, fNIRS has attracted increasing attention as an accessible and cost-effective alternative. fNIRS measures changes in the concentrations of oxygenated hemoglobin (OxyHb) and deoxygenated hemoglobin (DeoxyHb) in the blood of the cerebral cortex using near-infrared light (Chance et al., 1993; Hoshi and Tamura, 1993; Kato et al., 1993; Villringer et al., 1993; Pinti et al., 2020; Ayaz et al., 2022). Based on the neurovascular coupling hypothesis, this technique enables the assessment of cortical activation by measuring oxygenation dynamics in the cerebral blood flow. Because fNIRS uses low-intensity near-infrared light, it is noninvasive, imposes fewer restrictions on the measurement environment, and allows a high degree of freedom in participant posture during data acquisition. Although fNIRS cannot measure activity from deeper cortical and subcortical structures, research is limited to cortical regions and has a lower spatial resolution than fMRI, its practical advantages make it particularly suitable as a research tool for investigating olfactory responses in healthy participants (Azuma et al., 2015; Gunasekara et al., 2022; Hirabayashi et al., 2024).

The human brain extracts various types of information from olfactory stimuli; however, the central processing pathways of the human olfactory system are not completely understood. A distinguishing feature of the olfactory system, compared to other sensory systems, is the presence of multiple primary olfactory cortices (Gottfried, 2010; Haberly, 2001). These include the anterior olfactory nucleus (AON), olfactory tubercle (TUB), frontal piriform cortex (FPC), temporal piriform cortex (TPC), amygdala, and subregions of the entorhinal cortex (Insausti et al., 2002; Goncalves Pereira et al., 2005; Milardi et al., 2017). These regions are situated within the deep brain. Olfactory stimuli are projected in parallel from the olfactory bulb to multiple primary olfactory areas located deep in the brain. The olfactory bulb sends direct projections to numerous structures simultaneously, suggesting the existence of parallel functional pathways within the olfactory system (Kauer, 1991; Savic et al., 2000; Haberly, 2001; Cecchetto et al., 2019).

**FIGURE 1:**
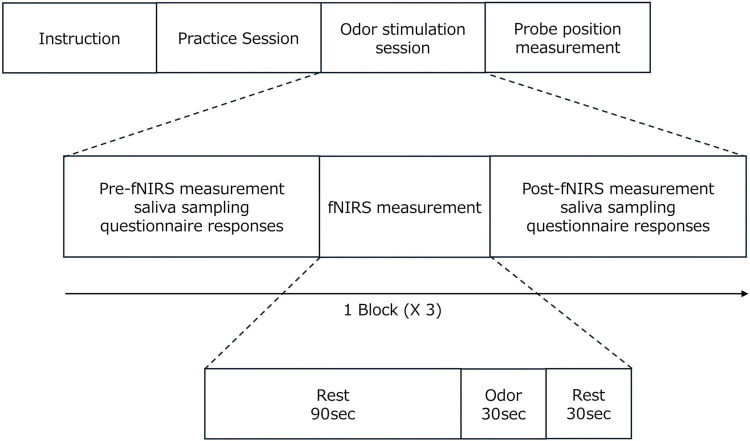
Schematic diagram of parallel functional pathways in the human olfactory system. The cerebral cortices interconnected by each functional pathway represent areas that can be assessed using functional near-infrared spectroscopy, with each region labeled as a region of interest.

Four functional pathways have been reported in non-overlapping brain regions that exhibit functional connectivity with the primary olfactory cortex (AON, TUB, FPC, and TPC) (Zhou et al., 2019). Each of the four functional pathways is connected to cortical regions and can be measured using fNIRS. Specifically, the AON connects to the orbitofrontal cortex (OFC), TUB connects to the frontal pole, FPC connects to the precentral gyrus, and TPC connects to the inferior frontal gyrus and left superior temporal gyrus (Figure 1). Although fNIRS studies often designate the OFC as the ROI, this study defined four cortical regions measurable by fNIRS as ROIs and analyzed the changes in OxyHb concentration within each region.

Employing these ROIs allows for a deeper understanding of their respective roles in olfaction (Zhou et al., 2019): AON-ROI: the initial step in forming a global percept of an odor; TUB-ROI: extracting social and emotional information from olfactory stimuli and mediating responses based on that information; FPC-ROI: planning olfactory-related behaviors, particularly those associated with feeding; TPC-ROI: linking olfactory information to language-related cortical areas.

Therefore, this study aimed to clarify whether objectively evaluating human body malodor is possible using fNIRS by defining regions of interest in the cerebral cortex based on functional pathways in the olfactory system. The human body malodor evaluated in this study was an artificial odor formulation developed to replicate the characteristic scalp odor observed in middle-aged women. This formulation is referred to as the middle-aged female scalp odor model (MFS model) (Kyuka et al., 2025). The MFS model odor was developed by the Mandom Corporation following consumer research that identified concerns regarding changes in body odor among women in their forties. Sensory evaluations conducted on participants in this age group revealed distinctive characteristics of the scalp odor, prompting a detailed analysis of its volatile components. Subsequent chemical analyses identified specific odor-causing compounds, which were incorporated into the model odor formulation. Further details are provided in Supplementary material.

To minimize interindividual variability (Yunpeng et al., 2020), a within-subject design was used in which participants evaluated three types of odors: the MFS model odor representing a human body malodor, isovaleric acid as a representative malodor, and lavender as a pleasant odor for comparison (Hirasawa et al., 2019). Although the order of odor presentation can influence perception, the primary aim of the study was to evaluate unpleasant body odors; therefore, to avoid potential bias introduced by pleasant odors, the presentation sequence was fixed such that unpleasant odors (isovaleric acid and the MFS model odor) were presented before pleasant odors. However, the order of the two malodors was randomized among the participants. To facilitate experiments in product development, such as cosmetics, odor stimuli were evaluated at an intensity such that participants could continue sniffing throughout the odor stimulation period without experiencing notable changes in mood or stress. Mood changes were assessed using a questionnaire, and stress was assessed using stress markers such as heart rate (Schubert et al., 2008; Kim et al., 2024), nasal tip temperature (Diaz-Piedra et al., 2019; Nakasai et al., 2022), fingertip blood flow (Ikawa and Karita, 2015; Seiyama et al., 2022), salivary cortisol, and salivary alpha amylase (Stegeren et al., 2008; Hirasawa et al., 2019).

## Materials and methods

### Participants

The participants were 18 healthy, right-handed, middle-aged Japanese individuals (nine women and nine men; mean age: 45.6 years, range: 41–49 years) recruited through a recruitment company.

A power analysis was performed before the study to determine the required sample size for detecting an interaction effect in a two-way repeated-measures ANOVA. Assuming an effect size of *f* = 0.25, an alpha level = 0.05, a power = 0.80, three groups, and a correlation among repeated measurements = 0.7, the required sample size was estimated to be 17 participants using G*Power version 3.1.9.7. 18 participants were recruited to allow for a buffer. All 18 participants were included in the final analysis.

The MFS model odor was reconstructed based on the chemical composition of characteristic odorants identified from scalp odor samples collected from women in their 40s. Because the aim of this study was to evaluate perceptual and neural responses to this specific model odor, participants were restricted to individuals in their 40s to ensure methodological consistency between the source population of the odor stimulus and the target population for evaluation.

All participants passed the olfactory test and distinguished between the standard odors. Olfactory function was screened using the T&T olfactometer (Daiichi Yakuhin Sangyo Co., Ltd.), a standardized olfactory test widely used for clinical and research purposes in Japan. Screening was performed using the 5–2 method in accordance with the Japanese Environment Agency Notification No. 63. In this test, two of five odor papers were impregnated with a standard odorant solution to approximately 1 cm from the tip, while the remaining three were treated with an odorless control solution. Participants were asked to correctly identify the two odorized papers. Only participants who correctly identified all five standard odorants at the panel selection reference concentration were considered to have normal olfactory function. No physician-based nasal examination was performed.

As this could have affected the test results, participants were prohibited from consuming caffeine or strong-smelling foods on the day of the test.

This study was reviewed and approved by the Mandom Ethics Committee, an independent ethics committee (Approval No. 108-22506; IRB No. 24000093; approval date: November 25, 2024). All procedures were conducted in accordance with the Declaration of Helsinki. Written informed consent was obtained from all participants before participation.

### Odor samples

The odors used were the MFS model odor developed to reproduce the scalp odor of middle-aged women as a body odor (Supplementary Table 1), isovaleric acid as a typical unpleasant odor, and lavender as a pleasant odor. The intensity of each odor was determined through preliminary testing by certified odor assessors. All odor samples were adjusted to an intensity level of 2.5 on a six-point odor intensity scale (0 = not perceptible, 5 = very strong) to ensure that participants could continue sniffing throughout the odor stimulation period without experiencing notable changes in mood or stress (Fuseda et al., 2025).

### Experiment design

**FIGURE 2:**
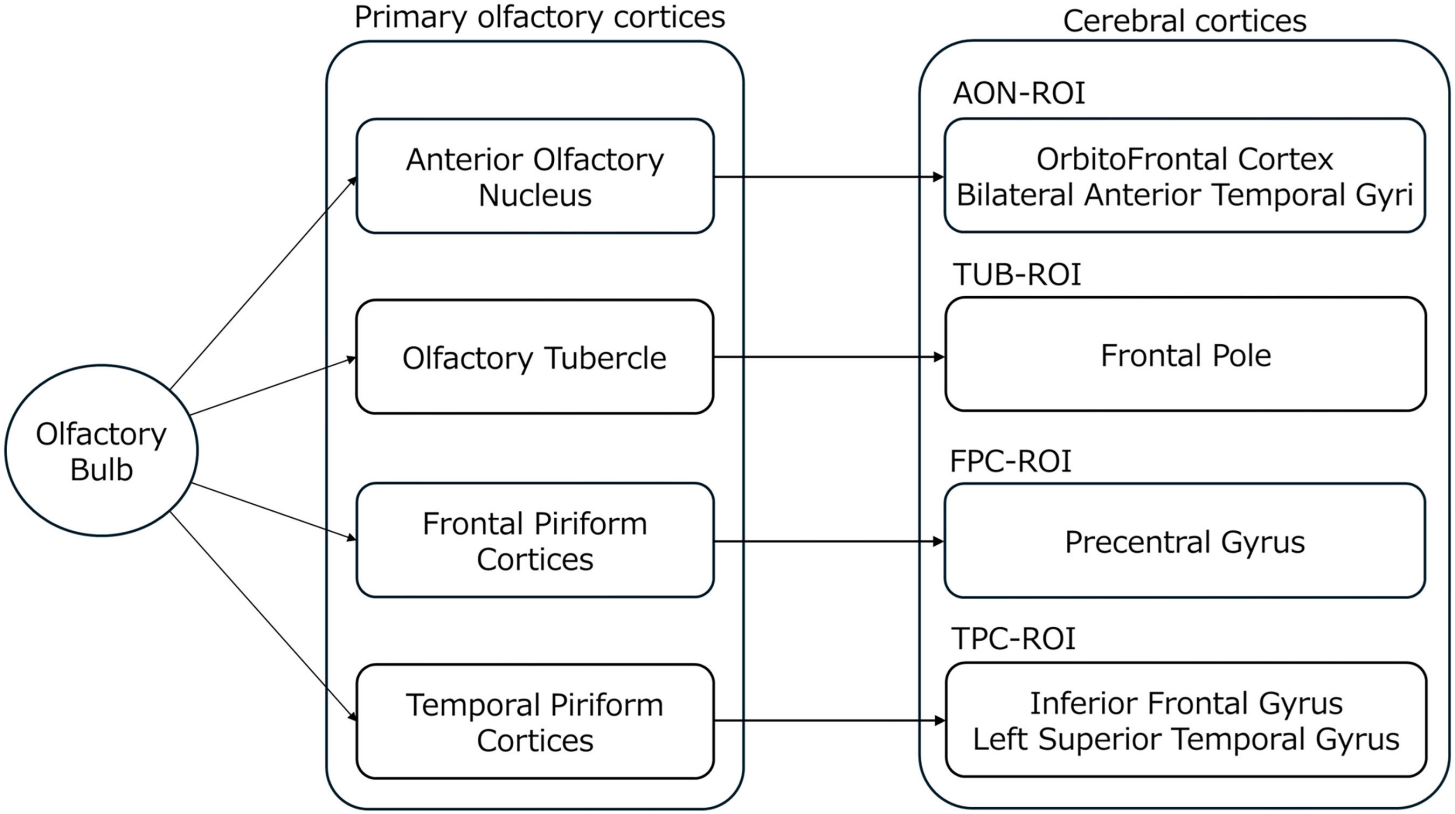
Experimental design. After one practice session without odor stimulation, three odor stimulation sessions were conducted with different types of odors.

The experimental design comprised three blocks: pre-fNIRS, fNIRS, and post-fNIRS blocks (Figure 2). After one practice session without odor stimulation, three blocks of experiments were conducted, each with a different odor.

As the primary aim of this study was to evaluate human body malodors, the presentation order of odor types was fixed as malodors (isovaleric acid, the MFS model odor), followed by a pleasant odor (lavender), to avoid potential bias induced by pleasant odors. The order of the two malodors, isovaleric acid and the MFS model odor, was randomized for each participant.

The participants completed questionnaires, and saliva samples were collected before and after each fNIRS measurement block was completed. The fNIRS measurement period comprised 90 s of rest, 30 s of odor stimulation, and 30 s of resting. Because the odor stimuli included both pleasant and unpleasant odors, spontaneous breathing patterns were expected to vary depending on odor valence and individual preference. It is known that odor valence can influence respiratory rate and depth, which may in turn affect autonomic and physiological responses, including heart rate variability, electrodermal activity, and neural signals. To reduce variability in physiological responses associated with uncontrolled respiratory fluctuations, participants’ breathing rate was controlled using a metronome during odor presentation. Based on pilot testing, the breathing rate was set to 35 breaths per minute. At this rate, participants were able to continuously inhale both pleasant and unpleasant odors throughout the 30-s presentation period without discomfort or excessive respiratory effort. Participants were instructed to synchronize their breathing with the metronome rhythm during each odor presentation trial. During the 30 s of odor stimulation, the experimenter presented the odors to the subject’s nose using a glass bottle to minimize the subject’s movement during fNIRS measurement. During the fNIRS measurement period, the heart rate, nasal tip temperature, and fingertip blood flow were simultaneously measured as stress markers.

After completing the three experimental blocks, the probe positions were digitized using a 3D magnetic spatial digitizer (Fastrak, Polhemus Inc., VT, United States) to account for variability due to interindividual differences in head size, and the fNIRS measurement channel locations were determined (Noah et al., 2015; Miguel et al., 2021).

### Questionnaire and saliva sample

To characterize the properties of the odor stimuli, the participants completed several questionnaires after the fNIRS measurements: odor intensity (six-point odor intensity scale, 0 = not detectable, 5 = extremely strong) (Fuseda et al., 2025), pleasantness/unpleasantness (visual analog scale, 0 = extremely unpleasant, 100 = extremely pleasant) (Dutheil et al., 2014), and odor preference (seven-point Likert scale, 1 = strongly dislike, 7 = strongly like). To assess mood changes induced by olfactory stimulation, participants completed a short-term mood scale measuring tension, depression, anger, confusion, fatigue, and vigor (five-point Likert scale, 1 = not at all, 5 = very much) before and after fNIRS measurements.

Saliva was collected using the swab method, and all samples were stored at −80°C in a frozen state until they were analyzed. Salivary cortisol levels were measured using the Expanded Sensitivity Salivary Cortisol Enzyme Immunoassay Kit (Salimetrics LLC, PA, United States), and salivary α-amylase levels were assessed using the Salivary α-Amylase Kinetic Enzyme Assay Kit (Salimetrics LLC, PA, United States) (Hirasawa et al., 2019).

### fNIRS data acquisition

**FIGURE 3:**
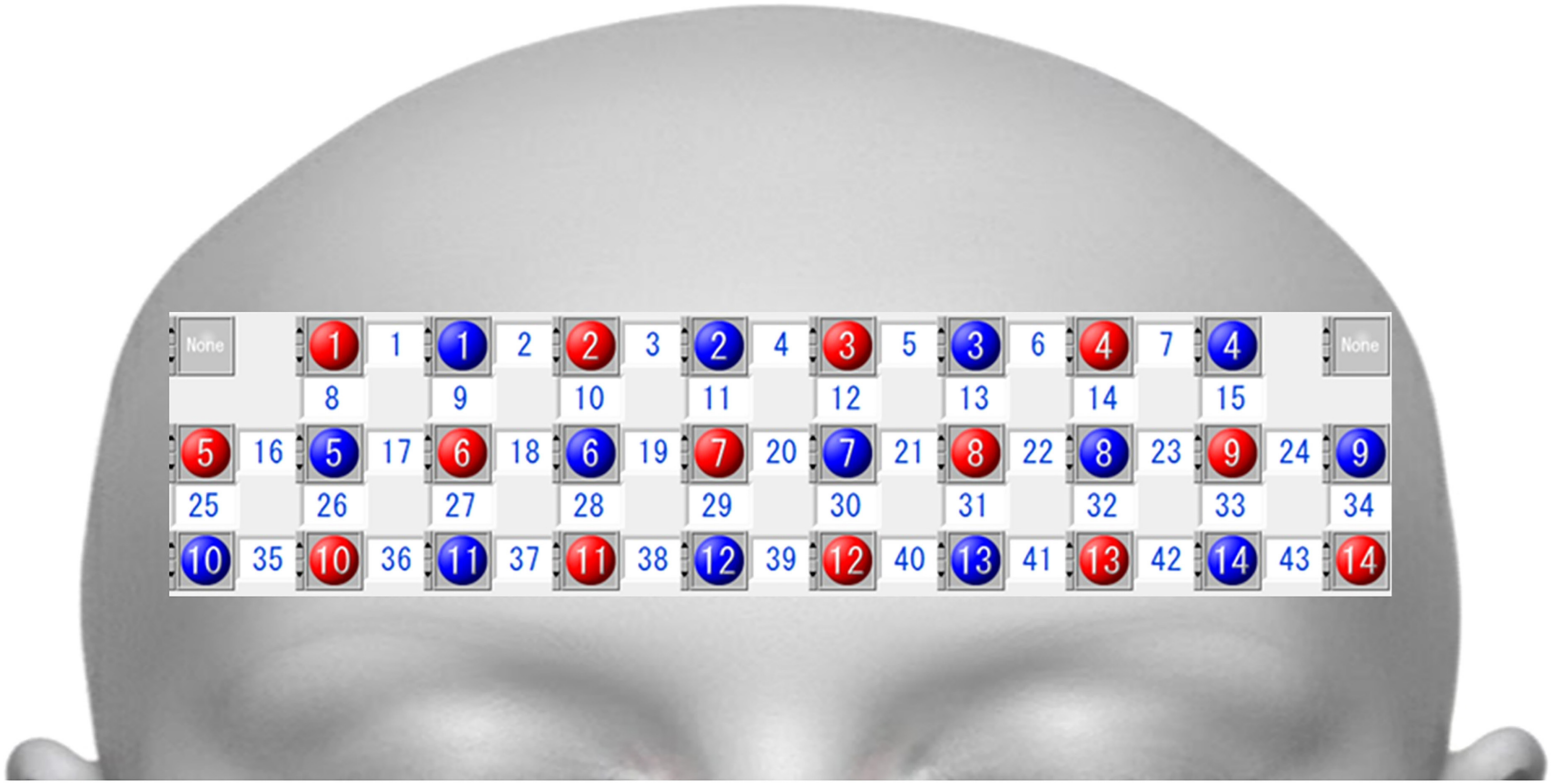
Spatial profile of functional near-infrared spectroscopy (fNIRS) channels. This figure shows the front view of the probe arrangement oriented toward the fNIRS channels. The detectors, light sources, and fNIRS channels are indicated by blue circles, red circles, and white squares, respectively.

Multichannel fNIRS (LABNIRS, Shimadzu Corporation, Kyoto, Japan) was used in the experiment. The system comprised 14 light sources and 14 detectors mounted on a probe holder, with a source–detector distance of 3 cm. The midpoint between each source and detector pair was defined as an “NIRS channel,” forming 43 channels (Figure 3). The probe holder was placed on the participant’s forehead such that channel 39 was located at Fpz, according to the international 10–20 system (Jasper, 1958).

Three wavelengths of near-infrared light (780, 805, and 830 nm) were used, and the data were acquired at a sampling rate of 22.2 Hz. The optical data were analyzed based on the modified Beer–Lambert law, and changes in OxyHb and deoxy-Hb concentrations were calculated in arbitrary units (mM⋅cm) (Noah et al., 2015).

### fNIRS data analysis

Preprocessing of the hemoglobin time-series data was performed for each of the 43 channels, taking into account motion artifacts, physiological noise, and contamination from scalp blood flow.

First, the presence of large motion artifacts was evaluated. During the measurements, participants maintained a seated posture, and continuous video recordings were used to confirm the absence of prominent body movements or head motion. Consistent with these observations, the fNIRS time-series data did not exhibit abrupt spike-like changes or pronounced baseline discontinuities typically associated with optode displacement or large motion artifacts. Therefore, no additional correction algorithms specifically targeting sudden non-physiological motion artifacts were applied.

However, motion-related and global hemodynamic fluctuations that may remain even under stable measurement conditions were addressed using the hemodynamic signal separation framework proposed by Yamada et al. (2012). In this framework, hemodynamic changes associated with body motion, postural adjustments, autonomic nervous system responses, cardiac activity, respiration, blood pressure fluctuations, and scalp blood flow are treated as components of a systemic signal. The method attempts to separate these components from cortical functional activity based on differences in the coupling patterns between OxyHb and DeoxyHb.

To reduce physiological noise, a low-pass filter with a cutoff frequency of 0.1 Hz was applied during preprocessing, attenuating high-frequency components related to cardiac activity as well as respiration-related fluctuations. In addition, the respiratory rate was experimentally controlled at approximately 35 breaths per minute to further reduce variability attributable to respiration. By combining temporal filtering with hemodynamic signal separation, we aimed to reduce the influence of physiological and superficial hemodynamic components on the extracted functional signals.

Contamination from scalp blood flow is a well-known challenge in fNIRS research. Two representative approaches have been reported to address this issue: short-separation channel regression and hemodynamic signal separation based on physiological characteristics. In the present study, we adopted the latter approach, following the framework proposed by Yamada et al. (2012). Although short-separation channels were not available, Yamada et al. (2012) reported that this framework yields functional components that show high consistency with cortical hemodynamic responses estimated using short-separation NIRS. Therefore, while this approach does not fully eliminate superficial and systemic influences, it is expected to assist in attenuating their effects at the hemodynamic level even in the absence of short-separation channels (Yamada et al., 2012; Kato et al., 2020; Hasegawa et al., 2022; Kawaguchi et al., 2024).

The hemoglobin time-series data for each of the 43 channels were preprocessed using a low-pass filter with a cutoff frequency of 0.1 Hz to eliminate noise. Functional brain components were extracted using hemodynamic modality separation algorithms (Yamada et al., 2012; Kato et al., 2020; Hasegawa et al., 2022; Kawaguchi et al., 2024).

Baseline correction was performed by setting the mean value of the 1-s period preceding task onset to zero for each dataset. Following these processing steps, the mean OxyHb concentration change during the 30 s odor stimulation period was calculated.

Based on information from a 3D magnetic spatial digitizer, the anatomical locations of all fNIRS channels for each participant were determined using the NIRS-SPM software (Ye et al., 2009). Anatomical labels with a probability of 50% or greater were defined as the anatomical locations corresponding to the fNIRS channels. The cerebral cortical regions measured using fNIRS and connected through four functional pathways were defined as the ROI. The anatomical locations and labels of the NIRS-SPM are listed in Supplementary Table 2.

For the AON ROI, one participant had missing fNIRS channels, and for the FPC ROI, two participants had missing channels. These ROIs were not excluded; instead, statistical analyses were performed with reduced sample sizes using the available data. The mean OxyHb concentration change in each ROI was calculated as the mean OxyHb concentration change in the fNIRS channels, with anatomical labels included in each ROI.

### Stress markers

During the fNIRS measurement period, electrocardiogram (ECG), nasal tip temperature, and fingertip blood flow were recorded as physiological stress markers. Cardiac potential was measured using an electrocardiogram device (HuME, Shimadzu Corporation, Kyoto, Japan) with electrodes attached according to the NASA lead configuration (+: xiphoid process, -: manubrium) at a sampling rate of 500 Hz. For the extraction of R–R intervals, ECG signals were preprocessed using standard filtering procedures. Specifically, notch filters were applied to remove power line noise at 50 and 60 Hz, followed by a bandpass filter with cut-off frequencies of 5–30 Hz. R-peaks were detected from the filtered ECG signals, and R–R intervals were calculated accordingly. Heart rate was calculated as the mean heart rate within each 30-s odor presentation period.

The nasal tip temperature was measured at a sampling rate of 10 Hz using a body-surface temperature sensor (AP-C050, Miyuki Giken Co., Ltd., Tokyo, Japan) attached to the tip of the nose and a data recording and analysis system (PowerLab System PL3508, ADInstruments Pty Ltd., NSW, Australia). The laboratory temperature was recorded simultaneously, and the nasal tip temperature data were corrected for fluctuations in the ambient temperature.

Fingertip blood flow was recorded at a sampling rate of 10 Hz using a laser tissue blood flow meter (OMEGAFLO FLO-C1, Omega Wave, Tokyo, Japan) with a sensor attached to the participant’s left index finger.

For each stress marker, the mean value was calculated for 30 s of the resting period before odor stimulation and 30 s during the subsequent odor stimulation, and the difference between these two periods was computed to quantify changes in stress markers associated with odor-induced brain activity.

## Results

### Subjective evaluation of odor samples

Based on the questionnaire responses assessing the characteristics of the odors, the pleasantness/unpleasantness and preference ratings indicated that the malodor samples—iso-valeric acid (pleasantness/unpleasantness mean = 16.6, preference mean = 2.1) and the MFS model odor (pleasantness/unpleasantness mean = 12.2, preference mean = 1.8)—were evaluated as unpleasant and disliked by the participants. In contrast, the pleasant odor sample of lavender (pleasantness/unpleasantness = 54.1, preference = 4.1) was rated as slightly pleasant and likable compared to the neutral value (pleasantness/unpleasantness = 50, preference = 4). Regarding odor intensity, iso-valeric acid (mean intensity = 4.1), the MFS model odor (mean intensity = 4.2), and lavender (mean intensity = 3.3) were all rated as relatively strong compared to the intensity level of 2.5 set by certified odor assessors.

Statistical analyses were performed using R software (version 4.5.2). Subjective ratings of pleasantness/unpleasantness, preference, and intensity were analyzed using repeated-measures two-way analyses of variance (ANOVA). The following within-subject factors were included: (1) odor type (isovaleric acid, MFS model odor, and lavender; hereafter referred to as “odor”), and (2) presentation order of the odor stimuli (iso-valeric acid and the MFS model odor). The significance level was set at α = 0.05.

For pleasantness/unpleasantness ratings, the ANOVA showed a significant main effect of odor type [*F*(2, 34) = 43.5, *p* < 0.001, η^2^ = 0.73], whereas neither the main effect of presentation order [*F*(1, 17) = 0.003, *p* = 0.96, η^2^ < 0.001] nor the interaction between odor type and presentation order [*F*(2, 34) = 0.21, *p* = 0.65, η^2^ = 0.002] was significant. Similarly, for preference ratings, a significant main effect of odor type was observed [*F*(2, 34) = 39.2, *p* < 0.001, η^2^ = 0.71], with no significant main effect of presentation order [*F*(1, 17) = 0.10, *p* = 0.75, η^2^ < 0.001] and no significant interaction [*F*(2, 34) = 0.03, *p* = 0.85, η^2^ < 0.001]. For intensity ratings, the main effect of odor type was again significant [*F*(2, 34) = 11.4, *p* < 0.001, η^2^ = 0.42], whereas neither the main effect of presentation order [*F*(1, 17) = 0.001, *p* = 0.92, η^2^ < 0.001] nor the interaction effect [*F*(2, 34) = 0.07, *p* = 0.79, η^2^ = 0.001] reached significance. Thus, significant main effects of odor type were found for pleasantness/unpleasantness, preference, and intensity.

Given the significant main effects of odor type, post hoc pairwise comparisons were performed using paired *t*-tests with Bonferroni correction. The results showed that both the MFS model odor and isovaleric acid were rated as significantly more unpleasant, less preferred, and more intense than lavender (corrected *p* < 0.05), whereas no significant differences were observed between the MFS model odor and isovaleric acid (corrected *p* > 0.05).

### ROI-based fNIRS data statistical analysis

#### Two-way repeated-measures analysis of variance (ANOVA)

For each ROI, a two-way repeated-measures ANOVA was conducted to examine the differences in the mean OxyHb concentration changes across the three odor stimuli. To investigate the effects of the type of odor stimulus on brain activity and the influence of the presentation order of the malodors, the same within-subject factors used for the analysis of subjective ratings were applied: (1) Odor type (iso-valeric acid, the MFS model odor, and lavender); and (2) Order of odor stimulus (iso-valeric acid and the MFS model odor). The significance level was set at *p* < 0.05.

In the TUB and FPC ROIs, neither the main effects of odor type nor presentation order were significant, and no significant interaction between odor type and presentation order was observed. In the AON ROI, neither the main effect of odor type nor that of presentation order reached statistical significance; however, a significant interaction between odor type and presentation order was detected. In contrast, in the TPC ROI, the main effect of odor type was significant, whereas neither the main effect of presentation order nor the odor type × presentation order interaction was significant. Overall, among the ROIs examined, a significant main effect of odor type was found only in the TPC ROI. Except for the interaction observed in the AON ROI, no significant effects of presentation order or interactions between odor type and presentation order were observed in the remaining ROIs (Table 1).

**TABLE 1:** Results of a two-way repeated-measures ANOVA examining the effects of odor type and presentation order on neural responses in each region of interest (ROI).

ROI	Effect	df	*F*	*p*-value	η^2^
AON	Odor	(2, 32)	1.10	0.35	0.057
	Presentation order	(1, 16)	0.003	0.96	0.000
	Odor × Presentation order	(2, 32)	6.26	0.02*	0.16
	Odor	(2, 34)	1.06	0.36	0.058
TUB	Presentation order	(1, 17)	0.06	0.81	0.002
	Odor × Presentation order	(2, 34)	2.64	0.11	0.007
FPC	Odor	(2, 30)	0.97	0.39	0.063
	Presentation order	(1, 15)	0.37	0.55	0.012
	Odor × Presentation order	(2, 30)	0.07	0.8	0.002
TPC	Odor	(2, 34)	4.01	0.03*	0.20
	Presentation order	(1, 17)	0.44	0.51	0.011
	Odor × Presentation order	(2, 34)	0.01	0.91	0.003

Effect sizes (η^2^) are rounded to three decimal places. *p ≤ 0.05.

#### *Post-hoc* analysis on the AON ROI and TPC ROI

In the AON ROI, where a significant interaction between odor type and presentation order was observed, simple main-effects analyses were conducted to further examine this interaction. When presentation order was held constant, a one-way repeated-measures ANOVA revealed a significant effect of odor type only when the MFS model odor was presented first [*F*(2, 14) = 4.82, *p* = 0.03, η^2^ = 0.13]. However, post hoc pairwise comparisons using paired *t*-tests with Bonferroni correction did not reveal any significant differences between individual odor conditions.

**FIGURE 4:**
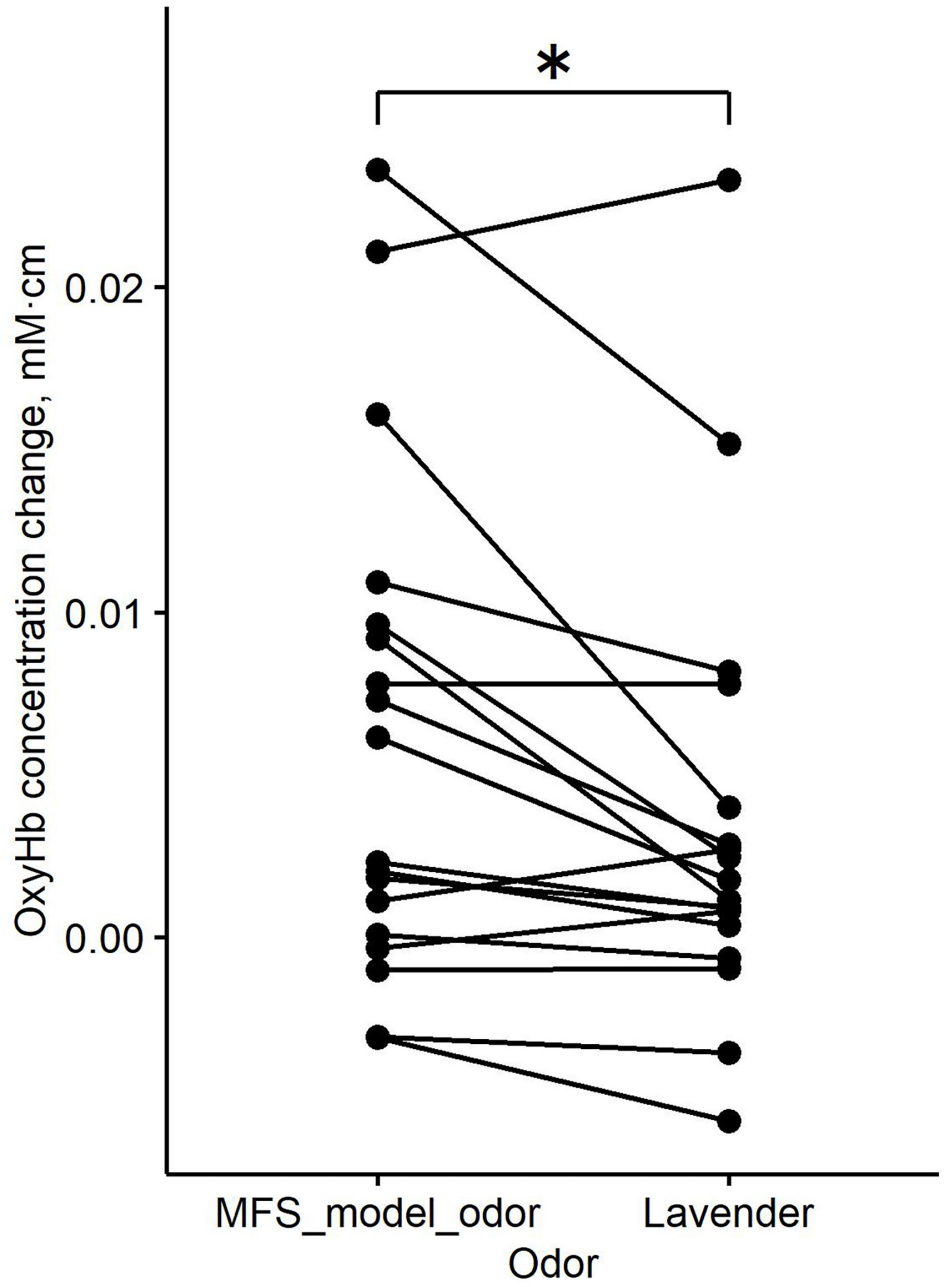
Pairwise connectivity diagram of OxyHb concentration changes within the TPC ROI under the MFS model odor and lavender stimulation conditions. * *p* < 0.05 (Bonferroni-corrected).

For the three odor stimuli in the TPC ROI, where a significant main effect of odor type was observed, *post-hoc* pairwise comparisons were conducted using paired t-tests with Bonferroni correction. The results showed that the increase in neural activity in the TPC ROI elicited by the MFS model odor was significantly greater than that elicited by lavender (mean difference = 5.013 × 10^−2^ mM⋅cm; corrected *p* = 0.02) (Figure 4).

#### Relationship between lavender and MFS model odor on TPC ROI brain activity

The OxyHb concentration change in the TPC ROI in response to the MFS model odor and lavender showed a strong positive correlation (*r* = 0.87). This strong correlation suggests that the magnitude of brain activity in the TPC ROI elicited by odor stimulation varies considerably across individuals, indicating the need for within-subject comparisons when evaluating differences in brain activity between odor stimuli.

#### Statistical analysis with subjective ratings as covariates

Given that subjective perceptual evaluations may influence olfactory-related neural activity, we conducted an additional analysis using a mixed-effects repeated-measures ANOVA to examine whether the observed differences in olfactory-related neural activity were attributable to subjective perceptual ratings. For neural responses in the TPC ROI, additional models were constructed in which each subjective rating—perceived intensity, pleasant/unpleasant and preference—was entered separately as a covariate. Odor type was treated as a within-subject factor, and participants were included as a random effect to account for repeated measurements. These analyses were to assess whether odor-related differences in TPC ROI activity persisted after statistically adjusting for each subjective evaluation that has been shown to influence neural responses to olfactory stimulation. In the TPC ROI, a significant difference in neural activity among the three odor conditions was observed when subjective evaluations were not included as covariates in the mixed-effects repeated-measures ANOVA. However, when subjective evaluations (perceived intensity, pleasant/unpleasant, or preference) were included as covariates, the previously observed main effect of odor type was no longer statistically significant.

#### Changes in temporary mood scales

To examine mood changes induced by odor stimuli, pre- and post-experimental scores on six subscales of the short-term mood measure (tension, depression, anger, confusion, fatigue, and vigor) were compared. Across all odor samples, the mean differences between the pre- and post-experimental scores ranged from –0.1 to 0.3 points, with all changes remaining below 1 point (Supplementary Table 3). These findings suggest that the odor stimuli used in this study were not sufficiently intense to elicit substantial mood changes. However, these results do not imply the absence of mood-related effects of odor stimulation.

#### Changes in stress markers

Noise was checked using ECG, nasal tip temperature, and finger blood flow data. For the ECG, a fast Fourier transform was applied to the raw signals, and data from two participants exhibiting prominent power line interference (60 and 120 Hz) were considered measurement failures and excluded from subsequent analyses. Across all odor conditions, the mean pre-post differences in heart rate ranged from –0.1 to –0.4 bpm (Schubert et al., 2008); nasal tip temperature differences ranged from –0.01 to –0.07°C (Diaz-Piedra et al., 2019); and finger blood flow differences ranged from –0.35 to –1.85 mL/min/100 g (Ikawa and Karita, 2015), which were small compared with those reported in previous studies (Supplementary Table 4). Therefore, the odor stimuli used in this study were unlikely to induce substantial stress responses. However, the primary aim of this study was to assess the effects of odor stimulation on brain activity, and the physiological measurements reported here represent pre-post differences associated with odor-related neural responses.

#### Changes in salivary cortisol and salivary alpha amylase

The mean pre-post differences in salivary cortisol levels measured before and after the fNIRS measurement ranged from –0.002 to 0.009μg/dL, indicating a small change. This finding is consistent with previous studies that reported that exposure to unpleasant odors does not substantially alter salivary cortisol levels (Hirasawa et al., 2019). In contrast, changes in salivary α−amylase levels showed large variability across both odor types and participants, with no consistent pattern, making interpretation difficult ( Supplementary Figure 1 and Supplementary Table 5).

#### Effects on stress markers and salivary measures

To evaluate the effects of odor type and presentation order on stress markers and salivary measures, repeated-measures two-way ANOVAs were conducted, as in the analysis of subjective ratings. The measures included heart rate, nasal skin temperature, fingertip blood flow, and salivary cortisol concentration. Salivary α-amylase was excluded because its large within-subject and within-condition variability rendered it unsuitable for condition-wise comparisons in the present experimental design.

No significant main effects of odor type or presentation order, nor significant interactions between these factors, were observed for heart rate, fingertip blood flow, or salivary cortisol concentration (all *p* > 0.05). For nasal skin temperature, neither the main effect of odor type nor the interaction was significant; however, presentation order showed a borderline effect (*p* = 0.05) (Table 2). Overall, none of the stress markers and salivary measures examined showed clear odor-dependent differences across conditions.

**TABLE 2:** Results of repeated-measures two-way ANOVAs for physiological and endocrine measures. Effect sizes (η^2^) are rounded to three decimal places.

Measure	Effect	df	*F*	*p*-value	η^2^
Heart rate	Odor	(2, 30)	1.03	0.38	0.07
	Presentation order	(1, 15)	0.01	0.93	0.000
	Odor × Presentation order	(2, 30)	0.04	0.84	0.001
	Odor	(2, 34)	1.96	0.16	0.10
Nasal skin temperature	Presentation order	(1, 17)	4.18	0.05	0.10
	Odor × Presentation order	(2, 34)	0.37	0.55	0.01
Fingertip blood flow	Odor	(2, 30)	2.01	0.15	0.11
	Presentation order	(1, 15)	0.08	0.78	0.002
	Odor × Presentation order	(2, 30)	0.48	0.49	0.013
Salivary cortisol	Odor	(2, 34)	2.32	0.09	0.12
	Presentation order	(1, 17)	0.58	0.45	0.01
	Odor × Presentation order	(2, 34)	0.31	0.38	0.07

## Discussion

The aim of the present study was to determine whether unpleasant body odor can be objectively evaluated using fNIRS by examining cortical processing related to the olfactory pathway. The results showed that, in the TPC ROI, the neural response to the MFS model odor was greater than that to lavender.

### Interpretation of neural activity mediated by subjective perception

To examine whether the observed differences in neural activity were attributable to subjective perceptual evaluations, subjective ratings were explicitly incorporated as covariates in the statistical models. When perceived intensity, pleasant/unpleasant and preference were each included in the model, the previously observed main effect of odor condition on activity in the TPC ROI was no longer significant. This finding suggests a close association between subjective perception and neural responses. This result does not imply that subjective evaluations functioned as independent confounding variables unrelated to odor conditions. Subjective ratings were elicited by each olfactory stimulus and systematically varied according to odor type, making it unlikely that they were causally independent of the experimental manipulation. Rather, subjective perceptual evaluations appear to reflect odor-specific experiences that are inherently linked to stimulus properties. Taken together, the present findings are more consistent with a mediational framework in which odor condition is associated with subjective perceptual experience, which in turn is associated with differences in neural responses. From this perspective, odor-related neural activity in the TPC ROI may be better understood as reflecting perceptual experience evoked by odor stimuli, rather than representing a direct effect of odor condition alone.

### Condition-specific differences in the TPC ROI

Condition-specific differences in the TPC ROI were observed only in the comparison between lavender and the MFS model odor, whereas no comparable difference was detected between lavender and isovaleric acid. According to Zhou et al., 2019, the TPC has been implicated in language-related processing. However, we do not intend to interpret the activity in the TPC ROI in the present study as a direct index of language processing *per se*. Instead, the observed TPC activity may reflect higher-order cognitive and control processes that can accompany olfactory perception, such as attentional engagement, cognitive load associated with odor evaluation, affective appraisal, or interpretive processing of odor stimuli. These processes may be differentially engaged depending on the qualitative characteristics of the odor and its subjective perceptual impact.

### Odor familiarity and cognitive processing

Lavender and isovaleric acid are relatively familiar odors with stable semantic and affective representations. As such, they may be processed in a more automatic manner, without requiring additional linguistic or conceptual exploration. In contrast, the MFS model odor can be regarded as a relatively unfamiliar stimulus. The condition-specific difference observed in language-related regions in the comparison between lavender and the MFS model odor suggests that processes such as semantic attribution, verbalization, or interpretive processing elicited by an unfamiliar odor may have differentially engaged these neural networks, potentially mediated by subjective perceptual experience.

### Divergence between subjective evaluations and neural activity

In the comparison between lavender and isovaleric acid, significant differences were observed in subjective ratings of perceived intensity and preference; however, no corresponding between-condition differences were detected in language-related neural activity. This dissociation suggests that differences in subjective evaluations do not necessarily translate into differences in linguistic or conceptual neural processing. These findings imply that neural differences observed in language-related regions cannot be explained solely by simple variations in hedonic valence or familiarity. Rather, they may reflect odor-specific perceptual and cognitive processes, as well as associated attentional, affective, and interpretive processing engaged by different odor stimuli.

### Odor intensity discrepancy

The discrepancy in odor intensity observed between expert ratings and participants’ subjective perceptions requires careful consideration from both neurological and perceptual perspectives. In the present study, participants’ intensity ratings should not be interpreted as pure sensory indices reflecting only physical odor concentration or peripheral sensory activity. Rather, a substantial body of olfactory research has shown that subjective odor intensity is closely associated with affective evaluation and attentional processes accompanying odor perception.

In particular, unpleasant odors are known to elicit heightened attention, emotional responses, and potentially interoceptive processing. Therefore, even when odors are judged by experts to be equivalent in physical concentration, it is methodologically plausible that odors with negative emotional valence are reported as subjectively more intense in a systematic manner. Accordingly, the marked discrepancy between participants’ intensity ratings (e.g., 4.1–4.2) and the expert-defined intensity level (2.5), despite strictly controlled stimulus preparation, can be interpreted within this framework. Similar reasoning can also be applied to differences in reported intensity values such as 3.3 vs. 4.2. These discrepancies should not be regarded solely as quantitative variations in sensory intensity. Instead, they may reflect qualitatively distinct perceptual states shaped by the emotional valence of the odor, expectancy, and cognitive evaluation processes. Accordingly, the observed mismatch in intensity ratings does not represent a methodological inconsistency; rather, it constitutes a meaningful perceptual difference that informs neural activity related to evaluative and interpretive processing of odor stimuli.

### Methodological considerations

First, the ROIs for fNIRS measurements were defined based on the cortical regions connected through four functional pathways. Although the central processing pathway of the human olfactory system is not yet fully understood, previous studies have shown that, unlike other sensory systems, the olfactory system comprises multiple primary olfactory cortices and cortical regions that are interconnected via four functional pathways (Zhou et al., 2019). In this study, these cortical areas were selected as ROIs because they represent the primary neural substrates involved in olfactory stimulation. However, it is important to note that fNIRS does not allow for direct measurement of neural activity in deep brain structures, including the piriform cortex. Accordingly, the activity observed within these cortical ROIs should not be interpreted as reflecting direct activation of the olfactory pathways themselves. Rather, these signals are more appropriately understood as indirect cortical representations related to downstream or associated processes involved in olfactory perception. From this perspective, the ROI approach adopted in the present study does not aim to directly measure the olfactory pathways per se, but instead provides a functional framework for indirectly examining cortical responses associated with olfactory perception.

Second, owing to individual differences in head size, we accurately determined the anatomical locations of all fNIRS channels for each participant based on the information obtained from a 3D magnetic space digitizer. As fNIRS channels frequently overlap with multiple anatomical regions, an anatomical label with a probability of > 50% was selected as the representative label for each channel. To ensure the validity of the fNIRS measurements, only ROIs containing channels that met this criterion for all participants were included in the analysis. Consequently, the ROIs with reliably assigned fNIRS channels across all participants were the TUB and TPC ROI. Although the AON ROI was not included in the analyses because one participant had no fNIRS channels localized to this region, the AON ROI might be included in future studies by adjusting the probe holder placement—for example, by positioning the Fpz landmark slightly above channel 39—to improve the coverage of the anatomical area (Jurcak et al., 2007).

Third, as an odor stimulation condition, the intensity of all odor samples was adjusted to a level at which the participants could continue sniffing throughout the odor presentation period without experiencing substantial changes in mood or stress. Consistent with this, the results of the temporary mood scale indicated that odor stimuli did not induce notable mood changes, and the physiological stress markers (heart rate, nasal tip skin temperature, and finger blood flow) showed differences that were too small to suggest meaningful stress induction. However, to link odor stimulation to brain activity, the odor presentation duration was set to 30 s, which is the standard task duration used in fNIRS studies. Compared with previous research on odors related to stress or aromatherapy (Saeki and Shiohara, 2001; Chien et al., 2011; Abdelhakim et al., 2020; Hedigan et al., 2013), the odor intensity used in this study may have been relatively weak, and the presentation duration was short. The stress markers analyzed here reflect pre-post differences within a 30-s window surrounding odor stimulation; therefore, these measures do not aim to assess autonomic responses to odors in the context of stress induction or relaxation.

Fourth, paced breathing was adopted to standardize respiratory conditions across participants. However, the increased respiratory rate (35 bpm) deviates from natural sniffing behavior and may have altered nasal aerodynamics. Computational fluid dynamics studies have shown that natural sniffing optimizes odorant delivery to the olfactory cleft compared with steady or paced breathing (Zhao et al., 2004; Zhao et al., 2006). Therefore, the paced breathing protocol employed here may have attenuated the effective concentration of odorants reaching the olfactory epithelium, a factor that should be considered when interpreting the present results. Although the elevated breathing rate was applied only briefly, it may have transiently increased alveolar ventilation, potentially inducing mild hypocapnia. Hypocapnia is a well-established cause of cerebral vasoconstriction and can modulate cerebral hemodynamics independently of neural activity. Because fNIRS signals are mediated by neurovascular coupling, such systemic vascular responses may have altered the baseline cortical hemodynamic state, representing a potential hemodynamic confound in the interpretation of neural responses.

Finally, lavender, a pleasant odor, was used as a reference condition, and the order of odor presentation was fixed such that unpleasant body odors were presented before the pleasant odor. The use of a reference odor was motivated by the expectation that the magnitude of brain responses to odor stimuli would vary substantially across individuals (Yunpeng et al., 2020). Consistent with this assumption, changes in Oxy-Hb concentration elicited by the MFS model odor showed a strong correlation with those elicited by lavender (*r* = 0.87), indicating marked interindividual variability in neural responses to odor stimulation. This finding underscores the importance of within-subject comparisons when evaluating neural responses to different odor stimuli. As a methodological consideration, because the primary objective of the present study was to evaluate responses to unpleasant body odors, the order of odor presentation was fixed as unpleasant-to-pleasant to minimize potential bias introduced by pleasant odors. Presenting unpleasant odors before pleasant odors was intended to reduce carryover effects associated with positive affect. Nevertheless, the possibility of sequential presentation effects or neural carryover warrants careful consideration. Cecchetto et al. (2019) reported that prolonged olfactory stimulation can induce persistent changes in resting-state functional connectivity after task completion, highlighting the importance of considering long-lasting effects of olfactory stimulation. However, the experimental conditions in their study differed substantially from those of the present study for stimulus duration and temporal structure. In Cecchetto et al. (2019), odor stimuli were presented continuously for approximately 9 min, followed by a 5-min resting-state measurement, whereas in the present study, each odor stimulus was presented for only 30 s, with intervals of more than 10 min between successive stimuli. This temporal design was intended to minimize neural carryover and adaptation effects across conditions. In addition, in the previous study reporting prolonged resting-state effects, lavender was the odor stimulus responsible for these effects, whereas in the present experiment, lavender was always presented last. Therefore, it is unlikely that residual effects related to lavender influenced neural responses to the other odor conditions (MFS model odor and isovaleric acid). Moreover, the order of presentation of the MFS model odor and isovaleric acid was counterbalanced across participants, further reducing the likelihood of systematic order-related bias. If the differences observed in the TPC ROI were primarily driven by sequential presentation or general neural adaptation, similar differences would be expected between the MFS model odor and isovaleric acid. However, significant differences were observed only in the comparison between the MFS model odor and lavender, whereas no significant differences were detected between isovaleric acid and lavender. This asymmetric pattern of results is difficult to explain solely by order effects or general neural adaptation. These considerations suggest that the differences observed in TPC ROI activity between the MFS model odor and lavender are unlikely to be fully accounted for by sequential presentation or neural carryover effects alone. Rather, they are more plausibly related to differences in subjective evaluation and interpretive processing associated with the odor stimuli. Although the influence of sequential presentation cannot be entirely ruled out, it is unlikely to represent the sole or primary factor underlying the main findings of the present study.

### Stress markers and respiratory control

In the present study, a comprehensive statistical evaluation was conducted for autonomic and endocrine stress markers. The results showed that heart rate, nasal skin temperature, fingertip blood flow, and salivary cortisol did not exhibit significant odor-dependent differences across the three odor conditions. These results indicate that the neural response differences observed in the TPC ROI are unlikely to be directly attributable to general stress responses or changes in overall arousal level. In particular, although nasal skin temperature showed a borderline effect related to presentation order, this effect was independent of odor type and therefore does not account for odor-specific neural activity patterns. Salivary α−amylase was excluded from the statistical analyses because of its large interindividual variability and sensitivity to measurement conditions. In addition, given the relatively short temporal structure of the olfactory stimulation paradigm, salivary α−amylase was considered unlikely to serve as a stable indicator for condition-wise comparisons in the present study.

Respiratory pace was controlled to reduce variability in autonomic responses arising from spontaneous breathing changes induced by odor pleasantness or unpleasantness, thereby increasing the validity of between-condition comparisons. Nevertheless, the possibility that respiratory control itself may influence neural and physiological activity cannot be entirely excluded. Accordingly, interpretations of heart rate data were restricted to mean values, and more detailed autonomic indices, such as sympathetic–parasympathetic balance or heart rate variability components, were not examined.

Taken together, physiological stress markers and respiratory control provide important contextual information regarding the background of the observed neural responses, but they are unlikely to represent primary mediators of the main effects observed in the present study. Instead, the neural activity differences are more plausibly related to perceptual and affective processes associated with subjective olfactory evaluations, such as perceived intensity and pleasant–unpleasant valence. This interpretation is consistent with and supported by the analytical approach adopted in this study, in which subjective perceptual ratings were explicitly incorporated as covariates.

### Scope of the study and generalizability

The primary aim of the present study was to show the validity of the proposed approach. To this end, we focused on a specific participant group—Japanese adults in their forties—and obtained fNIRS data that enabled the objective evaluation of unpleasant body odors. However, the relatively small sample size (*N* = 18) imposes certain limitations on statistical power and the generalizability of the findings. Although a priori power analyses were conducted to ensure adequate sensitivity for detecting interaction effects, the restricted sample size may have limited the ability to detect small effects, particularly in ROI-specific analyses and *post hoc* comparisons. Accordingly, the results of the present study should be interpreted with caution. To establish more robust and generalizable conclusions, future replication studies employing larger samples will be necessary.

No comparative analyses based on participant gender were conducted in the present study. This is because the primary objective was to examine differences in brain activity and subjective evaluations across odor conditions, and the sample size and experimental design were not planned to adequately assess effects related to these characteristics. Furthermore, menstrual cycle phase in female participants was neither recorded nor controlled, and therefore potential cycle-related fluctuations in olfactory perception cannot be entirely excluded. Nevertheless, because the analyses relied on within-subject comparisons across odor conditions, the influence of interindividual variability associated with these factors is considered to be at least partially reduced.

Future studies incorporating a broader range of participant characteristics, including different age groups (Mitro et al., 2012) and cultural backgrounds (Ayabe-Kanamura et al., 1998; Majid, 2021; Arshamian et al., 2022), are expected to provide more comprehensive and generalizable insights into olfactory processing. Moreover, building on the findings of the present study, the use of carefully designed, targeted questions may further deepen our understanding of the role of olfactory information in interactions among language-related brain regions. However, because providing participants with prior expectations may alter neural activity patterns, any questionnaires administered during experiments should be designed with particular care to minimize unintended biases. In conclusion, this study demonstrated that fNIRS can be used to objectively evaluate malodor in the human body in relation to cortical processing along the olfactory pathway. Further research using fNIRS holds substantial potential for providing additional objective information about olfactory perception and deepening our neurobiological understanding of malodors in the human body. These insights may be applied to the development of cosmetics and related products, ultimately contributing to improved comfort in living and working environments and helping reduce stress and dissatisfaction among individuals concerned with body odor.
