# Supplementary material for: Evaluation of human body malodor using functional near-infrared spectroscopy with regions of interest based on functional pathways in the olfactory system
Source: Front Neurosci. 2026 May 5;20:1808067. doi: 10.3389/fnins.2026.1808067 (PMC13183561; doi:10.3389/fnins.2026.1808067)
Supplement: Supplementary file 1 [file Data_Sheet_1.docx]

Supplementary Material

**Supplementary Material Table 1.** Ingredient list of middle-aged woman's scalp model odor.

| ingredient | ppm(v/v) |
| --- | --- |
| Butanal | 50 |
| 2-methylpropanal | 1 |
| 2-Butenal | 0.1 |
| 3-Methylbutanal | 50 |
| Pentanal | 10 |
| 2-Methylbutanal | 1 |
| Hexanal | 15 |
| Octanoic acid | 1,000 |
| Nonanoic acid | 1,000 |
| Decanoic acid | 1,000 |
| Diacetyl | 0.5 |

**Supplementary Material Table 2.** Relationship between regions of interest and their corresponding anatomical labels.

| ROI name | Primary olfactory cortex | Anatomical location of the cerebral cortex | Anatomical labels |
| --- | --- | --- | --- |
| AON ROI | Anterior Olfactory Nucleus | Frontal Orbital Cortex | Frontal_Sup_Orb_L, Frontal_Sup_Orb_R, Frontal_Mid_Orb_L, Frontal_Mid_Orb_R,　 Frontal_Inf_Orb_L, Frontal_Inf_Orb_R |
| TUB ROI | Olfactory Tubercle | Frontal Pole | Frontopolararea |
| FPC ROI | Frontal Piriform Cortex | Precentral Gyrus | Precentral_L, Precentral_R |
| TPC ROI | Temporal Piriform Cortex | Inferior Frontal Gyrus, Left Superior Temporal Gyrus | Frontal_Inf_Oper_L, Frontal_Inf_Tri_L, Temporal_Sup_L |

**Supplementary Material Table 3.** Mean changes on the Temporary Mood Scale before and after experiment for each odor condition. Values represent mean ± SD. Difference indicates After − Before.

| Subscale | Odor sample | Before-experiment | After-experiment | Difference |
| --- | --- | --- | --- | --- |
| Tension | Isovaleric acid | 5.0 ± 2.6 | 5.6 ± 2.8 | 0.6 ± 1.4 |
|  | MFS model odor | 5.1 ± 2.1 | 5.8 ± 2.3 | 0.7 ± 2.0 |
|  | Lavender | 5.0 ± 2.7 | 5.7 ± 3.1 | 0.7± 1.8 |
| Depression | Isovaleric acid | 3.8 ± 1.4 | 4.0 ± 1.6 | 0.2 ± 1.2 |
|  | MFS model odor | 4.1 ± 1.6 | 4.1 ± 1.7 | 0 ± 0.6 |
|  | Lavender | 3.8 ± 1.4 | 3.7 ± 1.4 | -0.1 ± 0.8 |
| Anger | Isovaleric acid | 3.4 ± 1.0 | 3.7 ± 1.4 | 0.3 ± 1.2 |
|  | MFS model odor | 3.4 ± 1.0 | 4.3 ± 2.1 | 0.8 ± 1.4 |
|  | Lavender | 3.5 ± 1.2 | 3.6 ± 1.1 | 0.1 ± 1.0 |
| Confusion | Isovaleric acid | 4.3 ± 1.4 | 4.5 ± 1.7 | 0.2 ± 0.7 |
|  | MFS model odor | 4.3 ± 1.4 | 4.6 ± 1.6 | 0.3 ± 1.1 |
|  | Lavender | 4.2 ± 1.3 | 4.3 ± 1.7 | 0.1 ± 1.2 |
| Fatigue | Isovaleric acid | 4.7 ± 1.9 | 5.2 ± 2.3 | 0.5 ± 1.3 |
|  | MFS model odor | 4.9 ± 2.1 | 5.4 ± 2.7 | 0.4 ± 1.1 |
|  | Lavender | 5.4 ± 2.8 | 5.2 ± 2.5 | -0.2 ± 0.5 |
| Vigor | Isovaleric acid | 7.6 ± 1.6 | 7.1 ± 1.7 | -0.6 ± 1.4 |
|  | MFS model odor | 7.6 ± 1.4 | 7.3 ± 1.6 | -0.3 ± 1.1 |
|  | Lavender | 7.3 ± 1.7 | 7.6 ± 1.9 | 0.3 ± 0.7 |

**Supplementary Material Table 4.** Mean changes on the Stress marker scores before and after experiment for each odor condition. Values represent mean ± SD. Difference indicates During − Pre.

| Item | Odor sample | Pre-exposure | During exposure | Difference |
| --- | --- | --- | --- | --- |
| Heart rate  (bpm) | Isovaleric acid | 77.3 ± 8.6 | 76.9 ± 8.4 | -0.4 ± 1.7 |
|  | MFS model odor | 75.3 ± 6.7 | 75.2 ± 7.1 | -0.1 ± 2.2 |
|  | Lavender | 74.9 ± 7.4 | 74.5 ± 7.9 | -0.4 ± 2.2 |
| Nasal tip temperature  (℃) | Isovaleric acid | 34.07 ± 0.62 | 34.06 ± 0.61 | -0.01 ± 0.10 |
|  | MFS model odor | 34.16 ± 0.52 | 34.09 ± 0.57 | -0.07 ± 0.12 |
|  | Lavender | 34.21 ± 0.52 | 34.16 ± 0.55 | -0.06 ± 0.11 |
| Fingertip blood flow  (ml/min/100g) | Isovaleric acid | 28.19 ± 5.76 | 26.34 ± 6.50 | -1.85 ± 3.67 |
|  | MFS model odor | 28.38 ± 6.11 | 27.06 ± 6.34 | -1.32 ± 2.00 |
|  | Lavender | 26.85 ± 5.92 | 26.49 ± 7.01 | -0.35 ± 3.58 |

**Supplementary Material Table 5.** Mean changes on salivary cortisol and salivary alpha amylase before and after experiment for each odor condition. Values represent mean ± SD. Difference indicates After − Before.

| Item | Odor sample | Before-experiment | After-experiment | | Difference |
| --- | --- | --- | --- | --- | --- |
| Salivary cortisol (μg/dL) | Isovaleric acid | 0.207 ± 0.084 | 0.216 ± 0.082 | | 0.009 ± 0.022 |
|  | MFS model odor | 0.212 ± 0.075 | 0.210 ± 0.082 | | -0.002 ± 0.025 |
|  | Lavender | 0.206 ± 0.112 | 0.213 ± 0.109 | | 0.007 ± 0.032 |
| Salivary alpha amylase (U/mL) | Isovaleric acid | 93.6 ± 66.2 | 81.7 ± 48.7 | | -11.9 ± 53.6 |
|  | MFS model odor | 71.5 ± 65.1 | 93.4 ± 71.7 | | 21.9 ± 51.4 |
|  | Lavender | 71.3 ± 56.1 | | 86.9 ± 53.8 | 15.7 ± 68.6 |


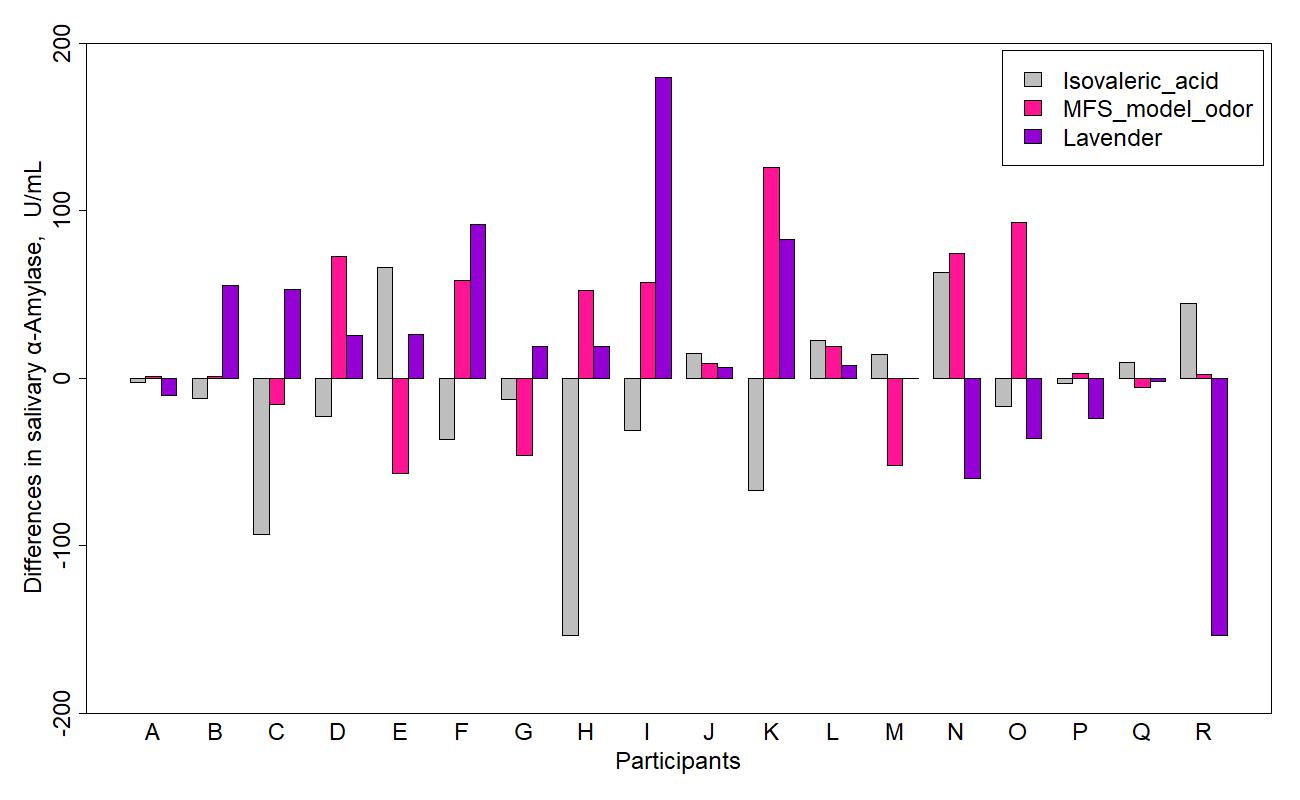


**Supplementary Material Figure 1.** Mean difference in salivary α-amylase before and after the experiments.
